# Endostatin expression in pancreatic tissue is modulated by elastase

**DOI:** 10.1038/sj.bjc.6602234

**Published:** 2004-11-16

**Authors:** R D Brammer, S R Bramhall, M C Eggo

**Affiliations:** 1 Division of Medical Sciences, University of Birmingham, Birmingham B15 2TT, UK; 2 Department of Surgery, Queen Elizabeth Hospital, Birmingham B15 2TH, UK

**Keywords:** endostatin, pancreatic cancer, elastase, angiogenesis

## Abstract

Pancreatic tumours are scirrhous, avascular tumours, suggesting that they may produce angiogenesis inhibitors that suppress the growth of the vasculature to the tumour and metastases. We have sought evidence for the angiogenesis inhibitor, endostatin, in normal and cancerous pancreatic tissue. Using Western blotting, we found mature 20 kDa endostatin in cancer tissue but not in normal tissue. Several endostatin-related peptides of higher mol wt were present in both tissues. Extracts from normal tissue were able to degrade exogenous endostatin, whereas extracts from cancer were without effect. Although the exocrine pancreas secretes inactive proenzymes of trypsin, chymotrypsin and elastase, their possible role in this degradation was examined. The trypsin/chymotrypsin inhibitor, *Glycine max*, did not prevent the degradation of endostatin by normal pancreatic extracts but elastatinal, a specific inhibitor of elastase, reduced the rate of degradation. Extracts of pancreatic tumours did not express any detectable elastase activity, but an elastase (*K*_m_ 1.1 mM) was expressed by extracts of normal pancreas. We conclude that endostatin is present and stable in pancreatic cancer tissues, which may explain their avascular nature, but that normal pancreatic tissue expresses enzymes, including elastase, which rapidly degrade endostatin. The stability of endostatin may have implications for its therapeutic use.

Pancreatic cancer is the fifth leading cause of cancer death in the UK, and despite advances in diagnosis, surgery and adjuvant chemotherapy, the prognosis remains poor (Bramhall *et al*, 1995; Jolly *et al*, 2002). Alternative methods of treatment therefore need to be investigated. Evidence exists that growth and resultant prognosis of pancreatic tumours is dependent upon microvessel density (Ellis *et al*, 1998). New capillaries sprout from existing blood vessels by a process known as angiogenesis. After adolescence, angiogenesis normally only occurs during reproduction, wound healing and states of chronic inflammation. It is a prerequisite for tumour growth and metastasis because without a sufficient blood supply, tumours rarely grow beyond a few millimetres in diameter (Folkman, 1990).

Angiogenesis is dependent upon both pro- and antiangiogenic factors (Folkman and Klagsbrun, 1987). One such antiangiogenic factor is endostatin. Endostatin is a 20 kDa fragment of the noncollagenous (NC-1) C-terminus of the basement membrane protein, collagen XVIII. Endostatin specifically inhibits endothelial cell migration, and induces cell cycle arrest and apoptosis *in vitro*, and *in vivo*, it reduces tumour growth (O’Reilly *et al*, 1997). Endostatin is now in clinical trials as an anticancer chemotherapeutic agent. Since collagen XVIII is a component of the vascular basement membrane, endostatin and endostatin-related proteins have been found in a variety of tissues (Saarela *et al*, 1998; Miosge *et al*, 1999). The occurrence of a broad range of endostatin-like fragments in tissues and serum suggests that collagen XVIII is sensitive to degradation at a number of sites by proteases (Standker *et al*, 1997; Sasaki *et al*, 1998; John *et al*, 1999; Ferreras *et al*, 2000).

Pancreatic tumours are scirrhous, avascular tumours, which suggests that the primary pancreatic tumour may produce an angiogenesis inhibitor, which suppresses the growth of the vasculature to the tumour and of metastases. Following surgical resection, removal of this angiogenesis inhibitor would result in removal of an inhibitory force acting upon the dormant micrometastases as elaborated by several authors (Folkman, 1990; 1995; Kirsch *et al*, 2000; 2004). The normal exocrine pancreas produces large amounts of proforms of the proteolytic enzymes trypsin, chymotrypsin and elastase, which are destined for the gastrointestinal tract. We hypothesised that pancreatic tissue contains collagen XVIII, and is therefore capable of producing endostatin from its precursor by proteolytic action of activated forms of these proteases or other proteases synthesised by the pancreas.

Evidence for the expression of collagen XVIII and endostatin in samples of both normal pancreatic tissue and pancreatic cancer was therefore sought. The regulation of endostatin production was investigated by studying endogenous protease action upon collagen XVIII and exogenous endostatin.

## METHODS AND MATERIALS

### Preparation of tissue lysates

Tissue samples were obtained at surgery and were frozen within 10 min of removal. Ethical approval was obtained for all tissue used. At all times, the tissue and the homogenates derived from the tissues were kept in an ice bath at 0°C to prevent warming and potential proteolysis. Approximately 1 cm^3^ sections of frozen normal pancreatic and pancreatic cancer tissue (*n*=5) were homogenised in 2 ml of Hank's balanced salt solution (HBSS) using a motorised homogeniser (Polytron). The homogenate was sonicated using five pulses of 30 s each and centrifuged at 15 000 **g** for 30 min. The supernatant was removed and stored at −20°C until use. In experiments examining expression of endostatin in tumour and normal tissues, 0.1% Triton X-100 was included to aid solubilisation from membranes. The pelleted, insoluble material was dissolved by boiling in 2% sodium dodecyl sulphate (SDS) in HBSS and stored at −20°C until use.

### Western blotting

A volume containing 100 *μ*g of protein was used and samples were heated to 60°C for 30 min in reducing sample buffer containing 100 mM 2-mercaptoethanol, 2% SDS and 62.5 mM Tris-HCl pH 6.8 before loading onto 12.5% SDS-polyacrylamide gels. Western blotting was performed as described previously using PVDF membrane (Hybond P, Amersham Pharmacia) (Michell *et al*, 1997). Primary antibody raised in rabbit to endostatin (Chemicon, 1 : 100 dilution) and horseradish peroxidase-labelled secondary antibodies (Santa-Cruz Ltd, 1 : 50 000 dilution) were used with enhanced chemiluminescence detection (ECL). The specificity of the endostatin primary antibody was confirmed by preabsorption to recombinant endostatin. No proteins were identified by ECL when the primary antibody was saturated with recombinant endostatin (data not shown).

### Stability of endostatin in normal pancreatic tissue and pancreatic cancer tissue

Aliquots of homogenates prepared in HBSS containing 100 *μ*g of normal or cancer tissue proteins were incubated at 37°C with 5 ng of recombinant endostatin (CN Bioscience) for periods of up to 20 h. Incubation was terminated at various time points by immersing the samples in an ice bath and adding reducing sample buffer. To test the thermal sensitivity of the reaction, 100 *μ*g of HBSS-soluble normal pancreatic extract was denatured by boiling and incubated with endostatin in the manner above. Analysis by Western immunoblotting was performed.

### Effect of protease inhibitors on stability of endostatin in tissue lysates

In total, 5 ng of endostatin was incubated for varying times up to 24 h at 37°C with HBSS-soluble pancreatic homogenate in the presence of either a combined trypsin–chymotrypsin inhibitor from *Glycine max* (soybean) (Sigma), at a concentration of 100 *μ*g inhibitor per 100 *μ*g total protein, or Elastatinal (Calbiochem, Germany), a specific, irreversible elastase inhibitor, at a concentration of 100 *μ*M.

### Elastase activity in normal and pancreatic cancer tissue

Activity of elastase in the pancreatic tissue lysate was evaluated with a colorimetric assay using *N*-methoxysuccinyl-Ala-Ala-Pro-Val P-nitroanilide as a chromogenic substrate as described by Bieth and Wermuth (1973). The molar extinction coefficient of the product formed was 8800, read at 410 nm in a 1 cm light path. Incubation was performed at room temperature on a 96-well microplate and the plate was read at intervals between 5 and 30 min up to a period of 24 h. The kinetics of the HBSS-soluble pancreatic tissue homogenates was evaluated and the assay was verified using purified neutrophil elastase (Sigma, UK). The results were analysed by plotting a Lineweaver–Burk curve to determine *K*_m_ and *V*_max_.

All experiments were performed in triplicate to confirm reproducibility.

## RESULTS

### Presence of collagen XVIII-endostatin in normal and cancerous pancreatic tissues

**Figure 1:**
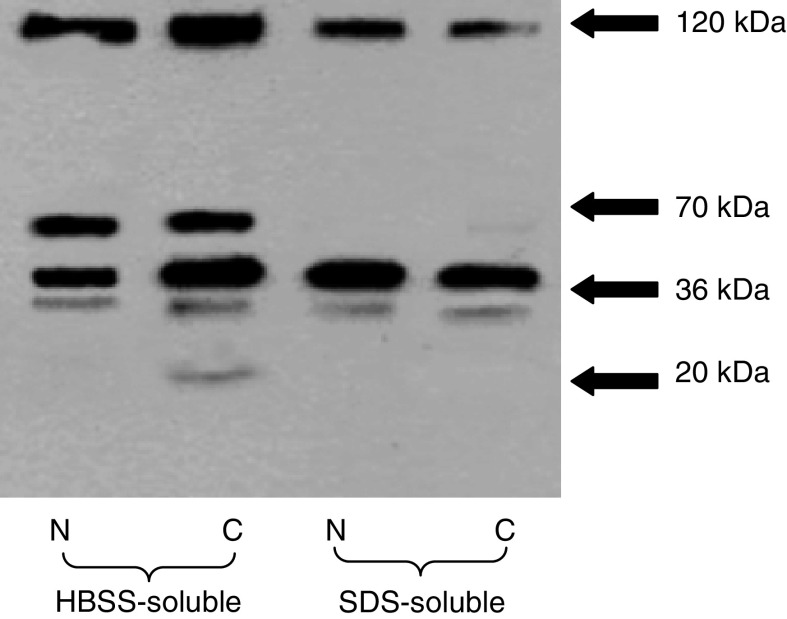
Western immunoblotting demonstrating the presence of endostatin-immunoreactive fragments in HBSS-soluble and SDS-soluble extracts from normal and cancer pancreas (N – normal pancreas, C – cancer pancreas).

Figure 1 shows a Western blot of extracts of proteins in the soluble fraction and the SDS-solubilised material from normal and cancerous pancreatic tissue, probed for endostatin. Immunoreactive bands are present at 120, 70, 36 and 20 kDa. The larger bands contain the endostatin fragment at their C terminus. The soluble fraction contains collagen XVIII (120 kDa), a 70 kDa fragment and the NC-1 fragment (36 kDa) in both normal pancreatic and pancreatic cancer tissue. The 20 kDa form of mature endostatin is present in the cancer tissue but not normal tissue. The SDS-solubilised fraction contains the 36 kDa fragment in both normal and pancreatic cancer tissue.

### Stability of exogenous endostatin in HBSS-soluble fractions from normal and cancer-derived pancreatic tissue

**Figure 2:**
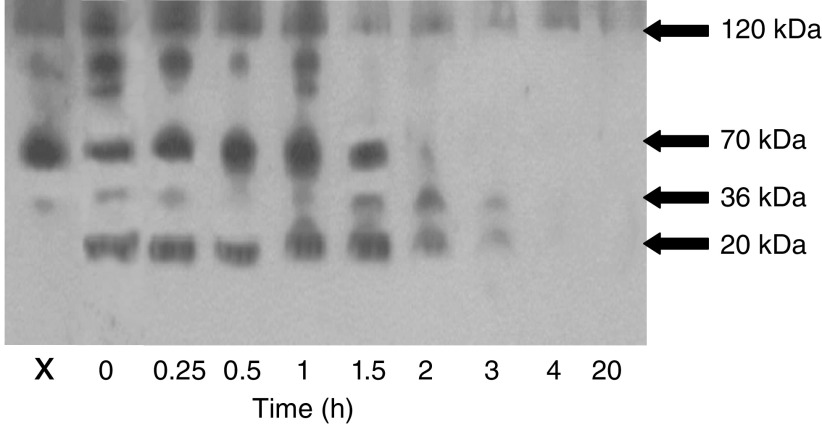
Western immunoblotting demonstrating that normal HBSS-soluble pancreatic homogenate degrades exogenous recombinant endostatin over time. A fixed amount of HBSS-soluble extract from normal pancreas was incubated with 5 ng of recombinant endostatin for the time periods shown. Recombinant endostatin migrates as a single band of 20 kDa. Sample x is the normal pancreatic homogenate without exogenous endostatin.

Recombinant endostatin was incubated with the HBSS-soluble fraction from normal pancreatic tissue and its stability determined by Western blotting. Figure 2 shows its stability with time. Degradation of collagen XVIII, endogenous endostatin-related fragments (70 and 36 kDa) and exogenous 20 kDa endostatin was seen at 2 h with complete degradation occurring by 4 h. The immunoreactivity of the endogenous endostatin-related fragment at 36 kDa was seen to diminish at 0.5 h and then increase to a maximum intensity at 2 h. This increase in intensity coincided with the loss of the immunoreactivity of the band at 70 kDa.

**Figure 3:**
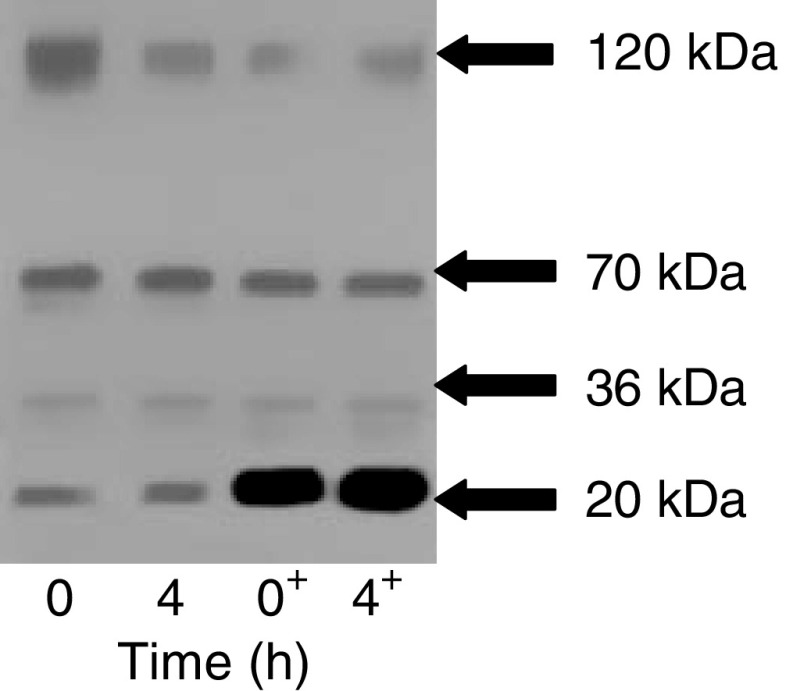
Western immunoblotting demonstrating that an HBSS-soluble extract from pancreatic cancer tissues does not degrade exogenous recombinant endostatin over 4 h. Exogenous endostatin (5 ng) was added to the samples in the lanes marked +.

The same experiment was repeated with HBSS-soluble extracts from pancreatic cancer tissue and the results are shown in Figure 3. Neither endogenous nor exogenous endostatin was degraded over time. Degradation of collagen XVIII and endostatin-related fragments (70 and 36 kDa) was not observed over a 4 h time period.

Heat treatment of the HBSS-soluble normal pancreatic extract by boiling prevented the degradation of endogenous endostatin-related fragments and exogenous 20 kDa endostatin (data not shown).

### Effects of protease inhibitors on endostatin degradation by HBSS-soluble extracts of pancreatic tissue

**Figure 4:**
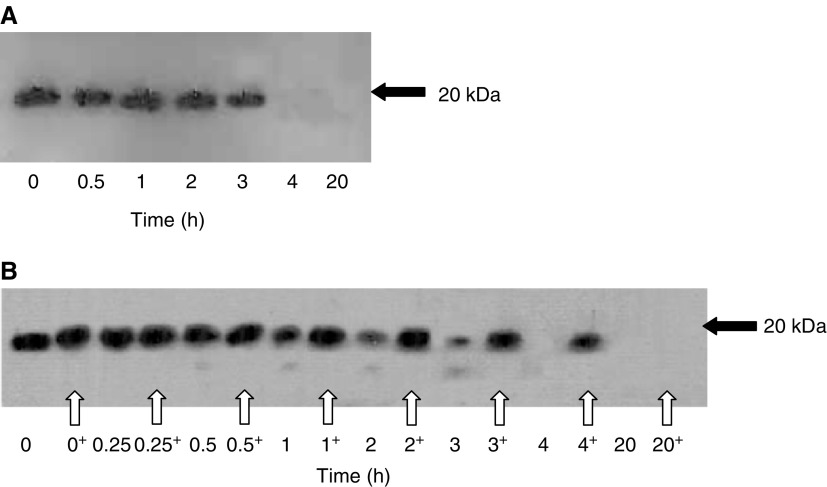
(**A**) Western immunoblotting demonstrating the effect of a chymotrypsin–trypsin inhibitor upon degradation of exogenous endostatin (5 ng) with time by normal pancreatic homogenates. (**B**) Western immunoblotting demonstrating the effect of the elastase inhibitor, elastatinal (10^−4^ M), on degradation of exogenous endostatin by normal pancreatic homogenates. Equal quantities of HBSS-soluble extracts of normal pancreatic tissue were incubated with 5 ng recombinant endostatin and in the absence or presence (marked^+^ and arrowed) of elastatinal for the indicated times.

To assess the potential contribution of endogenous pancreatic proteases, trypsin and chymotrypsin, to the degradation of exogenous endostatin, we performed the same experiment described in Figure 2 in the presence of a trypsin–chymotrypsin inhibitor in excess. When endostatin was incubated with HBSS-soluble extract from normal pancreatic tissue and the inhibitor, its degradation was not prevented (Figure 4A). By 4 h, all immunoreactive endostatin added to the sample had disappeared. The effects in Figure 2 were therefore unlikely to be due to the presence of endogenous active trypsin and chymotrypsin.

The effects of the elastase inhibitor, elastatinal, on the degradation of exogenous endostatin by an HBSS-soluble extract from normal pancreatic tissue are shown in Figure 4B. In the absence of elastase inhibitor, complete degradation of endostatin was seen by 4 h. In the samples with elastase inhibitor added (bold-type arrows), endostatin remained un-degraded at 4 h. By 20 h, when the experiment was terminated, endostatin had however been degraded.

### Elastase activity in extracts from normal and cancerous pancreatic extracts

**Figure 5:**
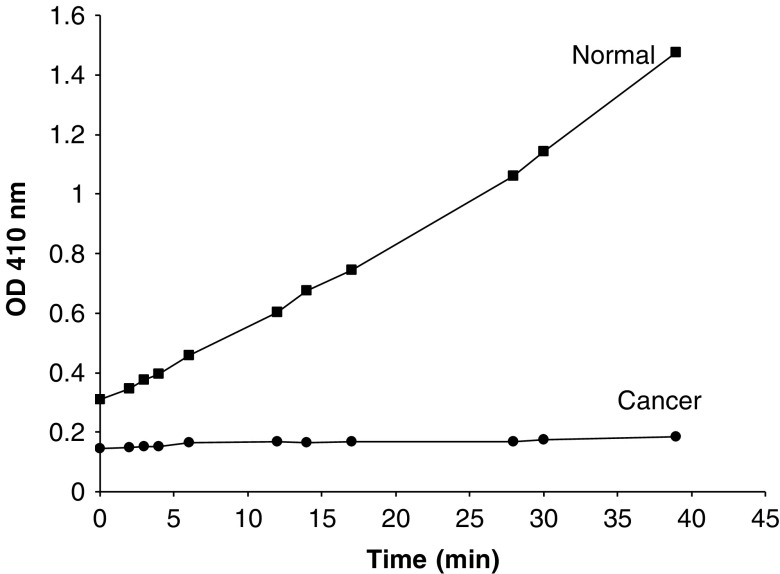
Elastase assay for normal and pancreatic cancer homogenates. HBSS-soluble extracts from normal and pancreatic cancer homogenates were incubated with *N*-methoxysuccinyl-Ala-Ala-Pro-Val P-nitroanilide as a chromogenic substrate as described by Bieth and Wermuth (1973). Incubation was performed at room temperature on a 96-well microplate and absorbance read at 410 nm.

Elastase activity, evaluated using the colorimetric assay described, is demonstrated for normal and pancreatic cancer tissue in Figure 5. There was a significant accumulation of product formed with time by the HBSS-soluble normal pancreatic tissue homogenate. In contrast, the pancreatic cancer tissue HBSS-soluble homogenate did not show any detectable product.

*K*_m_ for the reaction was calculated to be 1.1 mM using a Lineweaver–Burk plot of 1/rate *vs* 1/substrate concentration. The slope is *K*_m_/*V*, the *y* intercept is 1/*V* and the *x* intercept is −1/*K*_m_.

## DISCUSSION

Using Western immunoblotting we found that mature 20 kDa endostatin is present in pancreatic cancer tissue. Endostatin-related fragments and collagen XVIII were also present in normal pancreatic tissues but mature 20 kDa endostatin was not detected. Collagen XVIII and the NC-1 fragments, but not endostatin, were found in the SDS-soluble fractions from both tissues and we conclude that they are not totally soluble in HBSS, consistent with membrane localisation. Whether these endostatin-related fragments have any biological activity is unknown; however, their presence in numerous tissues and in serum suggests a potential role as does the fact that fragments of the structurally related collagen XV inhibit angiogenesis (Marneros and Olsen, 2001; Sasaki *et al*, 2002).

One explanation for the absence of endostatin in normal pancreatic tissue is that endogenous endostatin can be degraded by pancreatic proteases released during the homogenisation process. We demonstrated, using exogenous endostatin, that the normal pancreas contains enzymatic activity capable of degrading both exogenous mature 20 kDa endostatin and endogenous collagen XVIII and endostatin-related fragments. Boiling the normal pancreatic tissue extract prevented degradation of exogenous endostatin, consistent with proteolytic action. We also assayed whether varying concentrations of pancreatic cancer extract could inhibit the degradation of endostatin by normal pancreatic tissue but found no inhibition (data not shown) indicating that the effects of pancreatic cancer tissue were not due to the synthesis of a protease inhibitor.

Trypsin–chymotrypsin inhibition had no effect upon the ability of normal pancreas to degrade exogenous endostatin. This suggests that these enzymes are either not present or remain in their inactive proforms. Our studies show that elastase inhibition partially prevents endostatin degradation. Pancreatic elastase secreted into the GI tract is a protease of broad specificity and is activated in the duodenum by trypsin. We determined *K*_m_ and *V*_max_ for the elastase activity in the HBSS-soluble extracts. *K*_m_ was 1.1 mM, which compares with a reported *K*_m_ of 6.2 mM for porcine pancreatic elastase and 0.14 mM for human leukocyte elastase (Castillo *et al*, 1979). Whether the elastase that degrades endostatin is the elastase secreted into the GI tract remains unclear at present. It is certainly possible that it is a different elastase. Our data also suggest that HBSS-soluble pancreatic tissue homogenates contain other proteases capable of acting alone or in concert with elastase to degrade endostatin. Ferreras *et al* (2000) demonstrated that cathepsins L, B, K and D were also capable of degrading endostatin.

In contrast to normal pancreatic tissue, elastase activity was not detectable in pancreatic cancer tissue homogenates, which may explain both why mature 20 kDa endostatin is present in pancreatic cancer tissue and why exogenous endostatin is not degraded by pancreatic cancer homogenates. Since endostatin-related fragments were present in both normal and cancer tissues, elastase would appear to be more important in endostatin degradation than in its production.

Endostatin and endostatin-related fragments have been found in a number of tissues, which is not surprising since collagen XVIII is known to be a structural component of blood vessel walls. Endostatin release will result in a disturbance in the balance between angiogenesis promotion and inhibition, at a local level within the existing vasculature. Endostatin is known to regulate endothelial cell adhesion, survival and migration (Sasaki *et al*, 1998; Dhanabal *et al*, 1999). Conceivably following cellular injury, cellular proteases are released, which may degrade collagen XVIII completely with only transient or no production of endostatin. In this state, proangiogenic factors remain in the ascendancy and angiogenesis would result. In the absence of cellular injury and death, proteases capable of degrading endostatin are reduced and local levels of endostatin begin to rise again, restoring the balance between pro- and antiangiogenic factors. We hypothesise that endostatin is produced by proteolytic degradation of collagen XVIII in the normal population at a baseline level and is regulated by protease action rather than being produced in bursts when the body needs to reduce angiogenesis.

In cases of malignancy, in order for metastasis to occur, the balance of angiogenic factors must be biased in favour of proangiogenic factors and in favour of invasion, mediated through proteases. Pancreatic tumour cell lines are known to produce a plethora of proteases (Kitamura *et al*, 2000) and unusually to express receptors for angiogenic growth factors of the VEGF family (Itakura *et al*, 2000). Endostatin may work by inhibiting the mitogenic effects of these growth factors on the tumour cells as well as the endothelial cells (Kim *et al*, 2002). If tumour cells produce proteases capable of releasing endostatin from collagen XVIII, or angiostatin from plasminogen (O’Mahony *et al*, 1998) but do not produce proteases that are capable of degrading these antiangiogenic factors, then the tumour may be invasive but angiogenesis and metastases will be limited. In our experiments, tissue samples had been obtained from patients in whom metastasis had not occurred and it could be argued that this was because of the presence of endostatin.

We showed that elastase activity was undetectable in pancreatic cancer tissue, which is the likely explanation for why endostatin was not broken down. Patients with pancreatic tumours frequently have elevated circulating immunoreactive elastase Hayakawa *et al* (1990) but because higher levels are associated with a better prognosis, we suggest that this is a marker of the differentiation of the tumour. This circulating elastase is likely to be the inactive proform of pancreatic elastase, which would not thus degrade endostatin. Conversely, pancreatic tumours expressing macrophage metalloelastase have a worse prognosis than those that do not (Balaz *et al*, 2002). In this instance, we suggest that the elastase is active and degrades endostatin so that angiogenesis can occur as well as promoting invasion.

Folkman popularised the concept that surgical removal of tumours might remove a source of antiangiogenic factors and influence tumour biology by allowing metastatic disease to grow (Folkman, 1990; 1995). Our data support this by showing the presence and the stability of endostatin in pancreatic tumours. Tsunemi *et al* (2003) revealed progression of pulmonary metastases and significantly decreased serum concentrations of endostatin following removal of a primary osteosarcoma. What biological advantage endostatin production gives to the primary tumour is open to conjecture. Conceivably, there is a survival benefit due to reduced exposure to immunosurveillance. Alternatively, endostatin production may be viewed as a host defence. When the tumour secretes proteases to aid invasion, endostatin production due to collagen XVIII expression in the matrix would limit tumour spread. Only when the tumour can produce proteases, which degrade antiangiogenic factors, can metastases occur.

The use of endostatin as an anticancer agent is an exciting thought. It is likely however, that to be most effective, extended suppression of the angiogenic process is required. Since proteases have been shown to be capable of degrading endostatin, both *in vitro* and *in vivo*, delivery of endostatin as a chemotherapeutic agent in the conventional sense may be difficult. Consistent with this, recent data from a mouse tumour model have shown that continuous intraperitoneal infusion is more efficacious than intravenous bolus injections (Kisker *et al*, 2001).

## References

[bib1] Balaz P, Friess H, Kondo Y, Zhu Z, Zimmermann A, Buchler MW (2002) Human macrophage metalloelastase worsens the prognosis of pancreatic cancer. Ann Surg 235: 519–527 11923608 10.1097/00000658-200204000-00010 PMC1422467

[bib2] Bieth J, Wermuth CG (1973) The action of elastase on *p*-nitroanilide substrates. Biochem Biophys Res Commun 53: 383–390 4736813 10.1016/0006-291x(73)90673-6

[bib3] Bramhall SR, Allum WH, Jones AG, Allwood A, Cummins C, Neoptolemos JP (1995) Treatment and survival in 13 560 patients with pancreatic cancer, and incidence of the disease, in the West Midlands: an epidemiological study. Br J Surg 82: 111–115 7881926 10.1002/bjs.1800820137

[bib4] Castillo MJ, Nakajima K, Zimmerman M, Powers JC (1979) Sensitive substrates for human leukocyte and porcine pancreatic elastase: a study of the merits of various chromophoric and fluorogenic leaving groups in assays for serine proteases. Anal Biochem 99: 53–64 394626 10.1016/0003-2697(79)90043-5

[bib5] Dhanabal M, Ramchandran R, Waterman MJ, Lu H, Knebelmann B, Segal M, Sukhatme VP (1999) Endostatin induces endothelial cell apoptosis. J Biol Chem 274: 11721–11726 10206987 10.1074/jbc.274.17.11721

[bib6] Ellis LM, Takahashi Y, Fenoglio CJ, Cleary KR, Bucana CD, Evans DB (1998) Vessel counts and vascular endothelial growth factor expression in pancreatic adenocarcinoma. Eur J Cancer 34: 337–340 9640218 10.1016/s0959-8049(97)10068-5

[bib7] Ferreras M, Felbor U, Lenhard T, Olsen BR, Delaisse J (2000) Generation and degradation of human endostatin proteins by various proteinases. FEBS Lett 486: 247–251 11119712 10.1016/s0014-5793(00)02249-3

[bib8] Folkman J (1990) What is the evidence that tumors are angiogenesis dependent? J Natl Cancer Inst 82: 4–6 1688381 10.1093/jnci/82.1.4

[bib9] Folkman J (1995) Angiogenesis inhibitors generated by tumors. Mol Med 1: 120–122 8529090 PMC2229937

[bib10] Folkman J, Klagsbrun M (1987) Angiogenic factors. Science 235: 442–447 2432664 10.1126/science.2432664

[bib11] Hayakawa T, Kondo T, Shibata T, Kitagawa M, Katada N, Kato K, Takeichi M (1990) Prospective trial for early detection of pancreatic cancer by elevated serum immunoreactive elastase. Gastroenterol Japan 25: 727–731 10.1007/BF02779187 1703975

[bib12] Itakura J, Ishiwata T, Shen B, Kornmann M, Korc M (2000) Concomitant over-expression of vascular endothelial growth factor and its receptors in pancreatic cancer. Int J Cancer 85: 27–34 10585578 10.1002/(sici)1097-0215(20000101)85:1<27::aid-ijc5>3.0.co;2-8

[bib13] John H, Preissner KT, Forssmann WG, Standker L (1999) Novel glycosylated forms of human plasma endostatin and circulating endostatin-related fragments of collagen XV. Biochemistry 38: 10217–10224 10441114 10.1021/bi990787+

[bib14] Jolly K, Cheng KK, Langman MJ (2002) NSAIDs and gastrointestinal cancer prevention. Drugs 62: 945–956 11929340 10.2165/00003495-200262060-00006

[bib15] Kim YM, Hwang S, Kim YM, Pyun BJ, Kin TY, Lee ST, Gho YS, Kwon YG (2002) Endostatin blocks vascular endothelial growth factor-mediated signaling via direct interaction with KDR/Flk-1. J Biol Chem 277: 27872–27879 12029087 10.1074/jbc.M202771200

[bib16] Kirsch M, Schackert G, Black PM (2000) Angiogenesis, metastasis, and endogenous inhibition. J Neurooncol 50: 173–180 11245277 10.1023/a:1006453428013

[bib17] Kirsch M, Schaeckert G, Black PM (2004) Metastasis and angiogenesis. Cancer Treat Res 117: 285–304 15015566 10.1007/978-1-4419-8871-3_17

[bib18] Kisker O, Becker CM, Prox D, Fannon M, D’Amato R, Flynn E, Fogler WE, Sim BK, Allred EN, Pirie-Shepherd SR, Folkman J (2001) Continuous administration of endostatin by intraperitoneally implanted osmotic pump improves the efficacy and potency of therapy in a mouse xenograft tumor model. Cancer Res 61: 7669–7674 11606410

[bib19] Kitamura N, Iwamura T, Taniguchi S, Yamanari H, Kawano MA, Hollingsworth K, Setoguchi T (2000) High collagenolytic activity in spontaneously highly metastatic variants derived from a human pancreatic cancer cell line (Suit-2) in nude mice. Clin Exp Metastasis 18: 561–571 11688961 10.1023/a:1011900818419

[bib20] Marneros AG, Olsen BR (2001) The role of collagen-derived proteolytic fragments in angiogenesis. Matrix Biol 20: 337–345 11566268 10.1016/s0945-053x(01)00151-2

[bib21] Michell NP, Langman MJ, Eggo MC (1997) Insulin-like growth factors and their binding proteins in human colonocytes: preferential degradation of insulin-like growth factor binding protein 2 in colonic cancers. Br J Cancer 76: 60–66 9218734 10.1038/bjc.1997.337 PMC2223789

[bib22] Miosge N, Sasaki T, Timpl R (1999) Angiogenesis inhibitor endostatin is a distinct component of elastic fibers in vessel walls. FASEB J 13: 1743–1750 10506577 10.1096/fasebj.13.13.1743

[bib23] O’Mahony CA, Seidel A, Albo D, Chang H, Tuszynski GP, Berger DH (1998) Angiostatin generation by human pancreatic cancer. J Surg Res 77: 55–58 9698533 10.1006/jsre.1998.5334

[bib24] O’Reilly MS, Boehm T, Shing Y, Fukai N, Vasios G, Lane WS, Flynn E, Birkhead JR, Olsen BR, Folkman J (1997) Endostatin: an endogenous inhibitor of angiogenesis and tumor growth. Cell 88: 277–285 9008168 10.1016/s0092-8674(00)81848-6

[bib25] Saarela J, Ylikarppa R, Rehn M, Purmonen S, Pihlajaniemi T (1998) Complete primary structure of two variant forms of human type XVIII collagen and tissue-specific differences in the expression of the corresponding transcripts. Matrix Biol 16: 319–328 9503365 10.1016/s0945-053x(98)90003-8

[bib26] Sasaki T, Fukai N, Mann K, Gohring W, Olsen BR, Timpl R (1998) Structure, function and tissue forms of the C-terminal globular domain of collagen XVIII containing the angiogenesis inhibitor endostatin. EMBO J 17: 4249–4256 9687493 10.1093/emboj/17.15.4249 PMC1170758

[bib27] Sasaki T, Hohenester E, Timpl R (2002) Structure and function of collagen-derived endostatin inhibitors of angiogenesis. IUBMB Life 53: 77–84 12049199 10.1080/15216540211466

[bib28] Standker L, Schrader M, Kanse SM, Jurgens M, Forssmann WG, Preissner KT (1997) Isolation and characterization of the circulating form of human endostatin. FEBS Lett 420: 129–133 9459295 10.1016/s0014-5793(97)01503-2

[bib29] Tsunemi T, Nagoya S, Kaya M, Kawaguchi S, Wada T, Yamashita T, Ishii S (2003) Postoperative progression of pulmonary metastasis in osteosarcoma. Clin Orthop 407: 159–166 10.1097/00003086-200302000-00024 12567143

